# Detection of urinary microRNA biomarkers using diazo sulfonamide-modified screen printed carbon electrodes†

**DOI:** 10.1039/d0ra09874d

**Published:** 2021-05-25

**Authors:** Daniel A. Smith, Kate Simpson, Matteo Lo Cicero, Lucy J. Newbury, Philip Nicholas, Donald J. Fraser, Nigel Caiger, James E. Redman, Timothy Bowen

**Affiliations:** Wales Kidney Research Unit, Division of Infection & Immunity, School of Medicine, College of Biomedical and Life Sciences, Cardiff University Heath Park Cardiff CF14 4XN UK bowent@cf.ac.uk; Cardiff Institute of Tissue Engineering and Repair Museum Place Cardiff CF10 3BG UK; School of Chemistry, College of Physical Sciences and Engineering, Cardiff University Cardiff CF10 3AT UK; Sun Chemical Ltd Midsomer Norton, Radstock Bath BA3 4RT UK

## Abstract

This paper describes a straightforward electrochemical method for rapid and robust urinary microRNA (miRNA) quantification using disposable biosensors that can discriminate between urine from diabetic kidney disease (DKD) patients and control subjects. Aberrant miRNA expression has been observed in several major human disorders, and we have identified a urinary miRNA signature for DKD. MiRNAs therefore have considerable promise as disease biomarkers, and techniques to quantify these transcripts from clinical samples have significant clinical and commercial potential. Current RT-qPCR-based methods require technical expertise, and more straightforward methods such as electrochemical detection offer attractive alternatives. We describe a method to detect urinary miRNAs using diazo sulfonamide-modified screen printed carbon electrode-based biosensors that is amenable to parallel analysis. These sensors showed a linear response to buffered miR-21, with a 17 fM limit of detection, and successfully discriminated between urine samples (*n* = 6) from DKD patients and unaffected control subjects (*n* = 6) by differential miR-192 detection. Our technique for quantitative miRNA detection in liquid biopsies has potential for development as a platform for non-invasive high-throughput screening and/or to complement existing diagnostic procedures in disorders such as DKD.

## Abstract 2

In this study we have developed an electrochemical microRNA biosensor sensitive to 17 fM and capable of detecting an established downregulation of urinary miR-192 in diabetic kidney disease patients.

## Graphical abstract

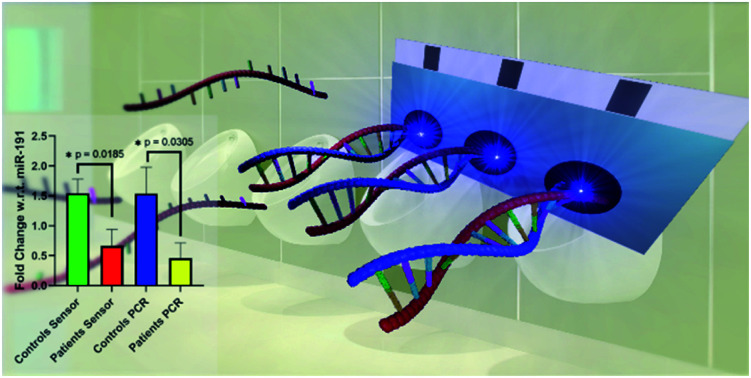

## 1. Introduction

MicroRNAs (miRNAs) are short single-stranded noncoding RNAs that regulate the expression of most mammalian protein coding genes. Aberrant miRNA expression profiles have been observed in major disorders including cancer, cardiovascular disease, atherosclerosis, diabetes and chronic kidney disease that requires treatment by dialysis or transplantation.^1–4^

While numerous previous studies have analysed the miRNA content of blood and/or tissue samples,^5,6^ we have developed RT-qPCR-based methods for precise quantification of miRNAs in a variety of liquid biopsies. In hypothermic machine perfusate, we showed that miR-21 may predict early renal transplantation outcomes.^7^ In peritoneal dialysis effluent we have identified neutrophil-derived miR-223 as a local biomarker of bacterial peritonitis,^8^ and shown increased miR-21 in peritoneal fibrosis.^9^

Detection of urinary miRNAs as disease biomarkers is particularly attractive, since this body fluid is readily collected without the need for invasive venepuncture or renal biopsy. We have identified panels of urinary miRNAs that predict delayed graft function following transplantation,^10^ and detect diabetic kidney disease (DKD).^11^ Our DKD patient data also showed significantly decreased urinary miR-192 in those suffering from DKD,^11^ supporting our previous findings from renal biopsy analyses.^12^

RT-qPCR is the current gold standard for miRNA quantification.^13^ We have used this technique in robust and accurate quantification of urinary miRNAs, and shown that these transcripts are stabilised by association with extracellular vesicles and/or argonaute 2 protein.^14^ However, RT-qPCR requires significant technical expertise, a drawback to implementation in routine testing at point-of-care.^13,15^

Methods using miRNA biosensors in blotting, fluorescence, and electrochemical procedures offer potentially attractive alternatives to RT-qPCR.^16–19^ A wide variety of electrochemical detection methods have been reported,^17^ including the use of aptamer-based probes,^20^ DNAzymes and nanoparticles.^21–23^ However, while these approaches offer improved sensitivity, their market potential is limited by requirements for complicated sensor fabrication and end-user expertise.

We developed a straightforward proof-of-concept electrochemical miRNA detection biosensor using a glassy carbon electrode (GCE) and DNA oligonucleotide with a sequence complementary to the target miRNA.^24^ This technique was more sensitive than RT-qPCR and discriminated between closely related oligonucleotide sequences, but was unsuitable for cost-effective clinical testing as the sensor required refabrication between analyses.^24^ Consequently, we report here biosensors prepared from inexpensive screen printed carbon electrodes (SPCEs). Electrochemical analysis through the use of SPCEs can be achieved using a portable potentiostat and consumables that are an order of magnitude cheaper than an RT-qPCR instrument, liquid handling system and reagents. SPCEs allow for customisations through printed electrode arrays for detection of multiple analytes or replicates, giving further benefits in cost reduction.

**Fig. 1:**
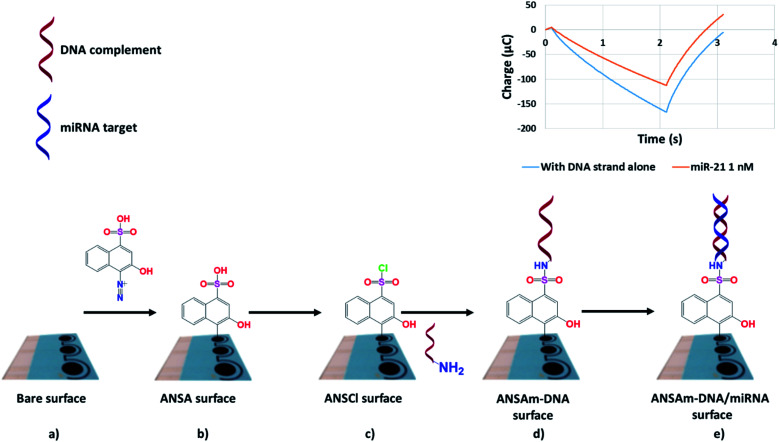
Fabrication of miRNA biosensors from screen printed carbon electrodes (SPCEs). The bare SPCE surface (a) is modified by electrochemical diazo sulfonic acid deposition to form an ANSA surface (b), which is further modified to a sulfonyl chloride (c) before addition of an amino terminated DNA strand complementary to the target miRNA, completing the biosensor (d). Hybridisation with the target miRNA (e) is detected electrochemically. For clarity, modification of only one of three working electrodes is shown.

Fig. 1 summarises SPCE-based biosensor fabrication, with SPCEs modified by deposition of diazotised naphthalene sulfonic acid derivative 4-amino-3-hydroxy-1-napthalene sulfonic acid (ANSA). The ANSA is then transformed into a sulfonyl chloride (ANSCl) before a 5′-amine-tagged miRNA-specific DNA oligonucleotide is attached *via* a sulfonamide linkage to complete the biosensor. The chemistry is easy to perform, with ANSA and oligonucleotides readily available from commercial suppliers. Biosensor readout is then carried out *via* reductive and oxidative chronocoulometry (throughout shortened to coulometry), obtained by measuring negative and positive potential sweeps using a ferri/ferrocyanide electrolyte, respectively, and readings are compared before and after miRNA-biosensor hybridization. The straightforward biosensor preparation and measurement is an advantage over more complex sensing platforms reported in the literature.^17,20–23^

Our novel SPCE-based biosensors are disposable, obviating the previous drawbacks using GCEs, but maintain high performance. Suitable for mass-production, they have potential for cost-effective parallel analyses of liquid biopsy miRNA biomarkers in the biochemistry laboratory and/or at point of-care.

## 2. Materials and methods

### 2.1 Materials and equipment

The following materials were obtained from the corresponding suppliers: DNA oligonucleotides, K_3_[Fe(CN)_6_], K_4_[Fe(CN)_6_], streptavidin peroxidase polymer (1 mg mL^−1^), chloroform, diethyl ether, ethanol, molecular biology grade water, from Merck (Watford, UK); tetramethylbenzidine (TMB) substrate solution, KCl, PCl_5_, NaNO_2_ and RT-qPCR reagents from Thermo Fisher Scientific (Gloucester, UK); RNA oligonucleotides from Integrated DNA Technologies (Leuven, Belgium); 4-amino-3-hydroxy-1-napthalene sulfonic acid (ANSA) from Fluorochem (Glossop, UK) and miRNeasy mini kit for miRNA extraction from Qiagen (Manchester, UK). Screen printed carbon electrodes (SPCEs) fabricated with proprietary ink formulations were supplied by Sun Chemical (Bath, UK). The optimised ink formulation product codes for SPCEs were: carbon (C2030519_P4), silver (C213016_D1) and dielectric (D2140114_D5). All DNA and RNA sequences used are listed in Fig. S1 of the ESI.†

Electrochemical experiments were performed using a PalmSens3 potentiostat (Alvatek, Tetbury, Gloucestershire, UK) with MUX8 multiplexer and combined reference and auxiliary over 3 working surfaces of the SPCEs. Data were analysed through PSTrace 5.4 supplied with the PalmSens3 potentiostat. Negative potential sweep (reductive) coulometry was carried out by applying a potential of 0.3 V for 0.1 s, 0.0 V for 2 s and finally 0.5 V for 2 s with measured intervals of 0.01 s; positive potential sweep (oxidative) coulometry by applying a potential of 0.0 V for 0.1 s, 0.3 V for 2 s, and 0.0 V for 3 s with measured intervals of 0.01 s. Differential pulse voltammetry experiments were performed between −0.3 V and 0.5 V at steps of 0.01 V, pulses of 0.05 V and 0.05 s at a scan rate of 0.05 V s^−1^. All electrochemical measurements were performed in 5 mM K_4_[Fe(CN)_6_)]/K_3_[Fe(CN)_6_] in 0.1 M KCl. The resulting data were plotted, and statistical analysis performed using GraphPad Prism® 9.

### 2.2 Biosensor fabrication

Prior to sensor fabrication the ANSA was diazotised *via* reaction with sodium nitrite in dilute hydrochloric acid. This helped ensure irreversible covalent linkage of the coating to the SPCE surface, which is more heterogeneous than the glassy carbon electrode our group used previously.^24^ Before use, the SPCEs were washed by submersion in 70% ethanol for 5 min and then for 10 min in water.

Firstly, 4-amino-3-hydroxy-1-napthalene sulfonic acid (48 mg, 11 mM) and sodium nitrite (17 mg, 14 mM) were dissolved in water (18 mL) and stirred to dissolution on ice. Once cooled, 0.1 M hydrochloric acid (2 mL, 10 mM final concentration) was added dropwise over 5 min, the solution was stirred for 30 min until development of a dark orange colour, and then neutralised according to universal indicator paper with small additions of sodium hydrogen carbonate. Without further purification, 500 μL aliquots were stored at −20 °C until needed.

**Fig. 2:**
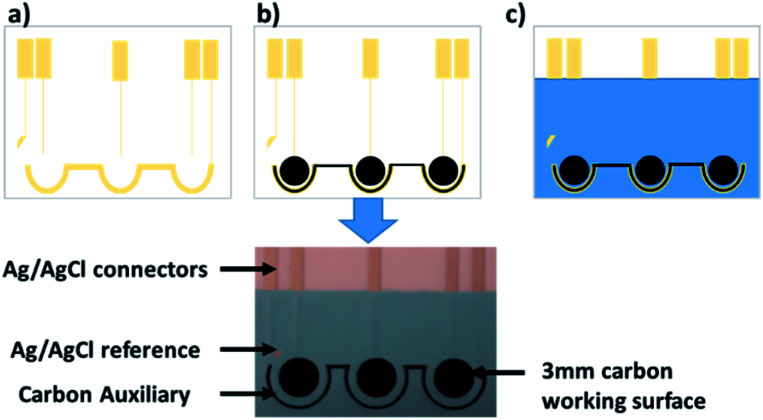
The design of the multi-surface SPCE used as the basis of our miRNA biosensor. The process of screen printing is summarised: (a) the silver/silver chloride tracers and reference are printed, (b) the carbon working and auxiliary surfaces are printed, and (c) the insulating dielectric ink is printed to define each working electrode surface.

Following diazo-ANSA synthesis, the SPCE (see Fig. 2) was rinsed with water for 5 min and then vortexed in water for a further 2.5 min to remove surface debris. The electrode was dried by shaking and connected to the potentiostat, 50 μL of diazo-ANSA was added to each surface and electrochemically deposited *via* 8 cycles of cyclic voltammetry between −0.5 V and 1.5 V at a scan rate of 20 mV s^−1^. After rinsing to remove residual diazo-ANSA, the electrode was vortexed for a further 2.5 min to remove any unbound diazo-ANSA, and then immersed in a solution of PCl_5_ (250 mg, 40 mM) in diethyl ether (30 mL) for 1 h to convert the sulfonic acid moiety to a sulfonyl chloride. The amino terminated DNA oligonucleotide with complementary sequence to the target miRNA (40 μL, 1 μM) dissolved in TM buffer (50 mM Tris HCl, 20 mM MgCl_2_, pH 8.0) was then denatured for 5 min at 90 °C, added to each working surface of the electrode and dried in a box oven at 80 °C for 1 h.

Once dry, the biosensor (DNA-modified SPCE) surfaces were rinsed with water for 5 min, vortexed in water for 2.5 min, and then analysed electrochemically *via* coulometry to obtain an initial baseline measurement corresponding to single-stranded DNA on the biosensor surface. The cyclic voltammetry, reductive coulometry and oxidative coulometry measurements at each stage are given in the ESI (Fig. S2–S5†).

### 2.3 Coulometry calibration plot for miR-21

The biosensors were rinsed to remove residual electrolyte, vortexed for 1 min to remove adsorbed electrolyte, and placed in a well of a Teflon block prior to addition of 800 μL of a TM-buffered miR-21 dilution between 10^−9^ and 10^−14^ M. The Teflon block was then placed on a rocking platform in a hybridisation oven at 55 °C for 1 h, after which the biosensor was removed and rinsed with TM buffer for 5 min, vortexed in TM buffer for 2.5 min and analysed electrochemically *via* coulometry. These final measurements of the DNA–RNA hybrid were compared to the initial measurement of the single-stranded DNA baseline described above, and the difference in the magnitude of the coulometric peak plotted against miR-21 concentration.

To obtain a miR-21 calibration plot, miR-21 biosensors were hybridised in triplicate as described above (*n* = 11). To account for any batch-to-batch variability, readings were pooled over different days from SPCEs generated in four separate print cycles *i.e.* across multiple printed cards. This is displayed in the error plotted as SEM. Triplicate readings for buffer alone (*n* = 6) were used as baseline values to determine the limit of detection.

### 2.4 Urine sample processing and electrochemical miRNA analysis

As described previously,^11^ urine samples were drawn from Wales Kidney Research Tissue Bank, University Hospital of Wales (Cardiff, UK) and ethical approval was granted by the Wales Kidney Research Tissue Bank Governance Committee.

Prior to analysis, urine samples were pre-treated using proteinase K and size exclusion spin filtration to remove proteins and other macromolecular interferents. A 480 μL urine aliquot was then mixed with 20 μL of proteinase K (20 mg mL^−1^) and CaCl_2_ (1 mg), and heated with shaking at 50 °C for 10 min. The urine was then pipetted onto a Millipore 10 kDa spin filter column and centrifuged at 14 000 rcf for 30 min at 4 °C. The filtrate (100 μL) was diluted with TM buffer (900 μL) and the electrode was immersed in the resulting solution at 55 °C for 1 h. Electrochemical detection data for miR-192 were normalised to those for miR-191 as we have detailed elsewhere.^11^

### 2.5 Urinary miRNA extraction and RT-qPCR analysis

Urinary miRNAs were extracted as we have described elsewhere.^14^ Extracted miRNAs were taken up in 50 μL of RNase-free water and stored at −80 °C until use. RT-qPCR analysis was carried out using a Thermo Fisher Scientific ViiA 7 System, with normalisation of miR-192 to miR-191 data.^14^

### 2.6 Uric acid interference

Sensors were submerged in TM buffer solutions containing 0, 250, 500, 750 or 1000 mg L^−1^ uric acid at 55 °C for 1 h, and then analysed electrochemically for evidence of interference. Then, sensors were resubmerged in uric acid solutions at the same concentration, but this time containing 10^−11^ M miR-21, at 55 °C for 1 h. Electrochemical analysis was used to compare miR-21-dependent surface responses.

### 2.7 Analysis of DNA deposition

For analysis of DNA deposition at the electrode surface, biosensors were fabricated (Fig. 1) using miR-21-specific DNA oligonucleotides that were either unlabelled or biotinylated. After drying at 80 °C for 1 h, sensors were rinsed with water for 5 min and vortexed for 2.5 min to remove unbound DNA. Incubation in a blocking solution (5 × SSC, 5% milk, 30 μL) was then carried out for 30 min at room temperature before rinsing in wash solution (5 × SSC). Then, streptavidin-HRP (1 μL, 1 mg mL^−1^) dissolved in TM buffer (1 mL) was added as individual droplets (30 μL) to sensors bearing labelled or unlabelled oligonucleotides complementary to miR-21 and left for 30 min at room temperature, after which the sensors were vortexed for 2.5 min in TM buffer as a first wash. Single sensors were then separated and washed in water for 30 min with shaking, rinsed with water, and placed in 2 mL microcentrifuge tubes with 250 μL of TMB substrate solution for 12 min. Addition of stop solution (H_2_SO_4_, 0.18 M) resulted in a yellow colour change that was analysed spectrophotometrically at 450 nm.

To investigate miRNA–DNA hybridisation at the electrode surface, biosensors specific for miR-21 or negative control miR-223 were fabricated. Sensors were then hybridised with a solution of biotinylated miR-21 RNA oligonucleotide (800 μL, 10^−7^ M) before rinsing and vortexing with TM buffer. Blocking, streptavidin addition (where appropriate), washing and analysis steps were then repeated as detailed above.

### 2.8 Atomic force microscopy (AFM) imaging

AFM imaging was performed with tapping mode (TM)-AFM in air at 293 K using a Nanoscope V instrument (Veeco, Plainview, NY, USA) type multimode 8. PPP-NCHR PointProbe Plus silicon SPM-sensor probes were used (Nanosensors, Neuchâtel, Switzerland; nominal resonance frequency 330 kHz, force constant 42 N m^−1^, length 125 μm) operating at a frequency of 321.5 kHz to image the surfaces through Scan Asyst-air mode, and data were analysed with WSxM software.

## 3. Results

### 3.1 Sensor design

To date, the majority of SPCE-based miRNA-specific biosensors have used single channel electrodes to detect singular miRNA species.^27–29^ These include a dual working surface system,^30^ and a method using DNA nanostructures on an array of 16 gold electrodes.^31^

By contrast, Erdem *et al.* demonstrated multiplexed miRNA detection using a system containing 16 active surfaces with shared counter and reference electrodes,^32^ and in one case used this set up to detect multiple miRNA species simultaneously.^33^ However these multiplex miRNA detection techniques required a laborious magnetic bead separation procedure to isolate the miRNA targets prior to analysis, and were tested on synthetic miRNA solutions and not patient samples.

In contrast to the GCEs we used previously,^24^ we report here our design of SPCEs incorporating 3 separate working surfaces (Fig. 1 and 2) that shared a combined auxiliary and reference electrode (Fig. 2). This design increased functionality and throughput, facilitating triplicate readings of a single miRNA species (Fig. 2). As an alternative, we are currently investigating simultaneous detection of up to 3 different miRNA species.

SPCE degradation was observed during chlorination *via* PCl_5_ in acetone,^24^ impairing sensor function and thereby introducing a source of variability (data not shown). Following extensive testing, chlorination using PCl_5_/diethyl ether for up to 3 h was possible without visible SPCE damage or decreased biosensor response (see below).

### 3.2 Sensor sensitivity

Coulometry was chosen as the electrochemical analysis method as it has been used successfully in our previous work and that of others,^24–26^ can be run in less than 5 seconds and is easily quantified by the magnitude of the scan peak which exhibits minimal background noise. Electrochemical impedance spectroscopy was explored as an alternative due to its ability to provide insight into the electrode-solution interface. However, difficulties in fitting a meaningful equivalent circuit model to the data from our SPCEs, and the short coulometric scan times and straightforward data processing led to the latter analysis being selected as the preferred method to take forward for our disposable SPCEs.

**Fig. 3:**
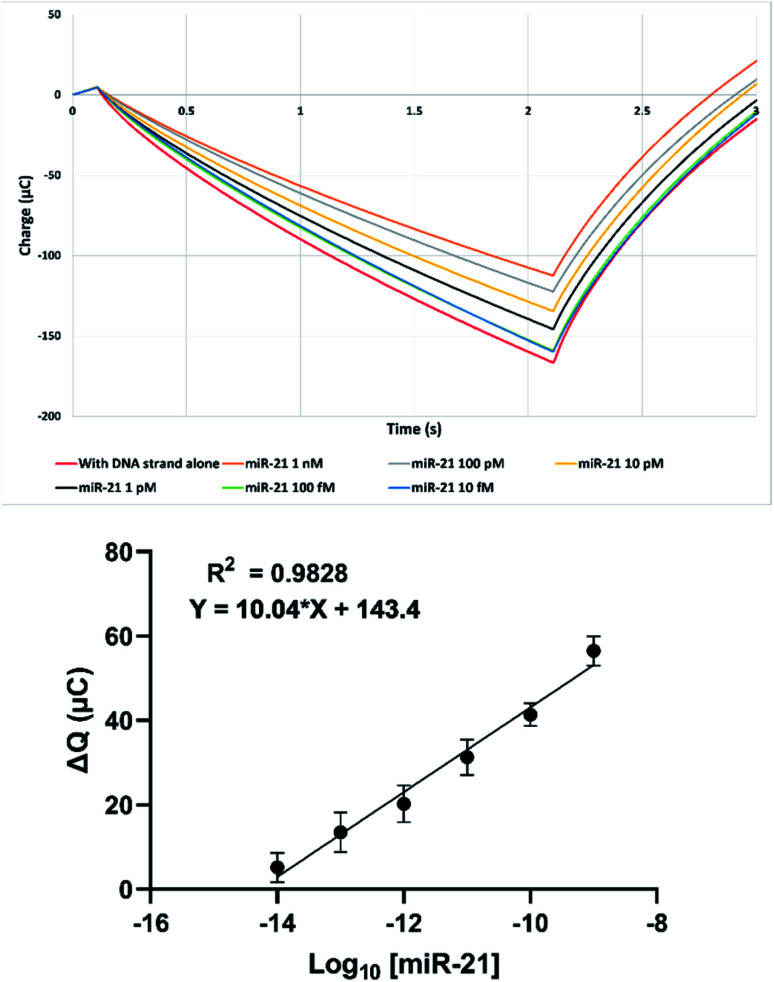
(Top) Example coulometric response following hybridisation of SPCE-biosensor with decreasing concentrations of miR-21. (Bottom) Reductive coulometric SPCE miR-21 biosensor response following hybridisation with varying concentrations of miR-21 over 4 separate experiments in [Fe(CN)_6_]^3−/4−^ in 0.1 M KCl. Data are expressed as mean ± SEM (*n* = 11).

Custom SPCEs were used for miR-21 biosensor fabrication as described above, and sensitivity was quantified using buffered miR-21 serial dilutions as shown in Fig. 3 TOP, Fig. S6 and S7 (ESI†). A linear response was observed between 10^−8^ M and 10^−14^ M (Pearson regression *R*^2^ = 0.98) that concurred with our previous GCE data.^24^ This value was obtained from pooling of data points taken over multiple sensor print cycles, thus including the effect of batch-to-batch variability in the error bars shown. An SPCE limit of detection of 17 fM compared favourably with our GCE value of 20 fM (Fig. 3 bottom, ESI Fig. S7†),^24^ and is on a par with other disposable miRNA electrochemical sensors in the literature.^34,35^ This limit of detection was calculated from 6 readings using a blank buffer solution containing no miRNA.

The calibration plot (Fig. 3) was produced from sensors taken over 4 separately printed cards of 9 electrodes each. Minor variations between SPCE replicates when plotting Δ*Q* against [miR-21], shown by the standard error of the mean (SEM) error bars, reflect card-to-card differences. Differential pulse voltammetry was also performed (ESI, Fig. S8 and S9†) to confirm, although this method showed greater variability so was not taken forward.

### 3.3 Urinary analysis

To the best of our knowledge, the typical absolute molar concentration range of miRNA species in urine have not been established definitively. Our previous report showed evidence for detection of urinary miR-21 from healthy controls in the pM concentration range,^24^ a range supported by luminescence-based detection.^36^ The 17 fM detection limit we report here is orders of magnitude more sensitive, and is therefore suitable for reliable qualitative urinary miRNA analysis.

**Fig. 4:**
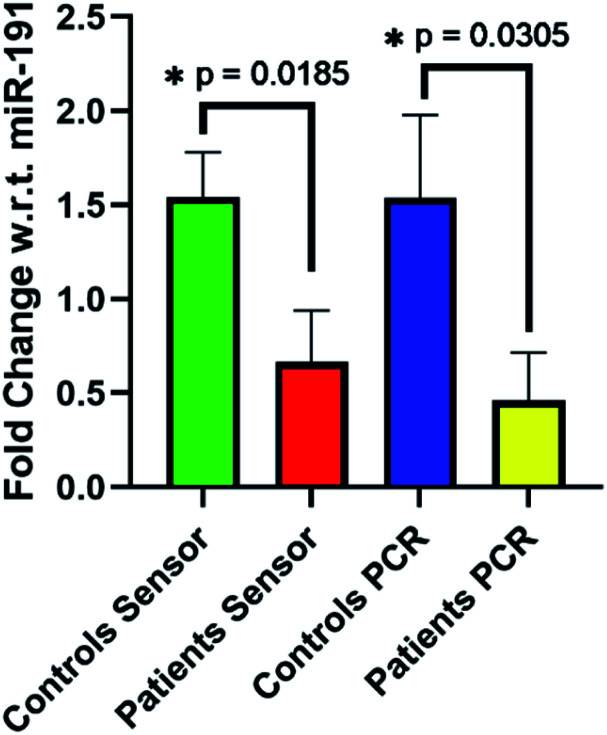
Reductive coulometry (Echem) and RT-qPCR (PCR) analysis of urinary miR-192 relative expression in DKD patients and control subjects. Data were normalised to miR-191 and are expressed as means ± SEM (*n* = 6).* *p* < 0.05.

To test the utility of our SPCE biosensors to detect DKD biomarkers, we analysed miR-192 in patient and control urine samples. We have shown previously that miR-192 abundance decreases in biopsy samples from DKD patients.^12^ Our electrochemical data were compared to the results of parallel RT-qPCR analysis, with relative expression data for miR-192 normalised to those for miR-191 as we have described elsewhere.^11^ For both electrochemical and RT-qPCR detection, significant differences in miR-192 detection were observed between unaffected individuals and DKD patients (Fig. 4). Using our sensor, miR-192 expression with respect to miR-191 fell from a 1.54 fold change in the control cohort to 0.66 fold change in the patients. This was in good agreement with RT-qPCR analysis of the same samples which showed a decrease from 1.54 fold in the controls to 0.46 in the patients. Similarly, oxidative coulometry (ESI, Fig. S10†) showed the same change in effect, albeit slightly more pronounced, dropping from a 2.15 fold change in controls to 0.54 in the patients.

We then prepared a serial dilution series from one control urine sample and carried out coulometric analysis for miR-21 to control for interference from contamination with macromolecules such as proteins that might exhibit non-specific high affinity binding to the electrode surface. The linear relationship observed suggested that there was no significant contamination (ESI, Fig. S11†).

The effect of interference by proteins and small molecules has been referred to in other literature.^36,37^ To avoid these issues of non-specificity, a full extraction procedure is often used to isolate the miRNA prior to analysis,^37–39^ whereas “pre-treatment free” approaches often use synthetic miRNA spiked-in to the urinary matrix which is then analysed.^36^ Our simple proteinase K treatment avoids laborious full extraction procedures such as those we describe herein prior to RT-qPCR analysis, but facilitates direct urinary miRNA measurement in a simple, potentially scalable protocol.

In our previous work, we investigated the direct analysis of untreated urine as a biological matrix.^24^ From this we determined that salt and urea do not interfere, however protein in the form of bovine serum albumin (BSA) was problematic. We therefore implemented the aforementioned urine pre-treatment of proteinase K incubation and filtration. In this research we have also investigated the impact of uric acid as another potential interferent. Uric acid is normally excreted in urine at a level of 250–750 mg over a 24 h period. We found that uric acid at or above the normal range resulted in no interference. No change in response was observed when biosensors were submersed in uric acid solutions in the absence of miR-21. None of the uric acid solutions prevented or interfered with the response of the same sensor to 10^−11^ M miR-21 (ESI, Fig. S12 and S13†).

### 3.4 Surface chemistry analysis

Biosensor-miRNA interaction was then characterised further. Streptavidin/biotin chemistry was used to provide further evidence for SPCE–DNA oligonucleotide attachment and oligonucleotide–miRNA binding (Table 1).

**Table 1:** *A*_450_ readings from TMB substrate and stop solution ± streptavidin-HRP (S-HRP) for (a) non-biotinylated and biotinylated miR-21 biosensors and (b) non-biotinylated miR-21 or miR-223 biosensors hybridised with 10^−7^ M 5′-bio-miR-21

Spectrophotometric analysis at 450 nm (*A*_450_)					
(a) Non-biotinylated or biotinylated (bio) miR-21 biosensors			(b) Non-biotinylated miR-21/miR-223 biosensors + 5′-bio-miR-21		
Comp-miR-21 − S-HRP	Comp-miR-21 + S-HRP	3′-Bio-comp-miR-21 + S-HRP	Comp-miR-21 − S-HRP	Comp-miR-223 + S-HRP	Comp-miR-21 + S-HRP
0.000^a^	0.010 ± 0.002	0.123 ± 0.014	0.000^a^	0.008 ± 0.003	0.091 ± 0.006

a Baseline response − streptavidin has been subtracted from the data, values are expressed as mean ± SD (*n* = 3). Oligonucleotides' details are provided elsewhere (ESI, Fig. S1).

Firstly, a miR-21 biosensor was prepared using non-biotinylated or biotinylated DNA oligonucleotides complementary to miR-21 (Table 1a). Streptavidin-HRP conjugate was applied to one non-biotinylated biosensor and one biotinylated biosensor. A further non-biotinylated biosensor was not exposed to conjugate. Following blocking, colour development by immersion in TMB substrate solution, and sulfuric acid stop solution, a >12 fold increase in signal was observed using the biotinylated oligonucleotide-based biosensor (Table 1a), the predicted outcome if oligonucleotide attachment was successful.

The specificity of miR-21 detection was then investigated by comparing hybridisation of biotinylated miR-21 with non-biotinylated miR-21 and miR-223 biosensors (Table 1b). Following exposure to biotinylated miR-21 RNA, streptavidin treatment and spectrophotometric analysis, a >11-fold signal increase was observed for the miR-21 biosensor compared to its miR-223 counterpart (Table 1b). These data demonstrated that the biosensor DNA oligonucleotide-miR-21 interaction was specific.

### 3.5 Surface tapping mode-atomic force microscopy (TM-AFM) imaging

**Fig. 5:**
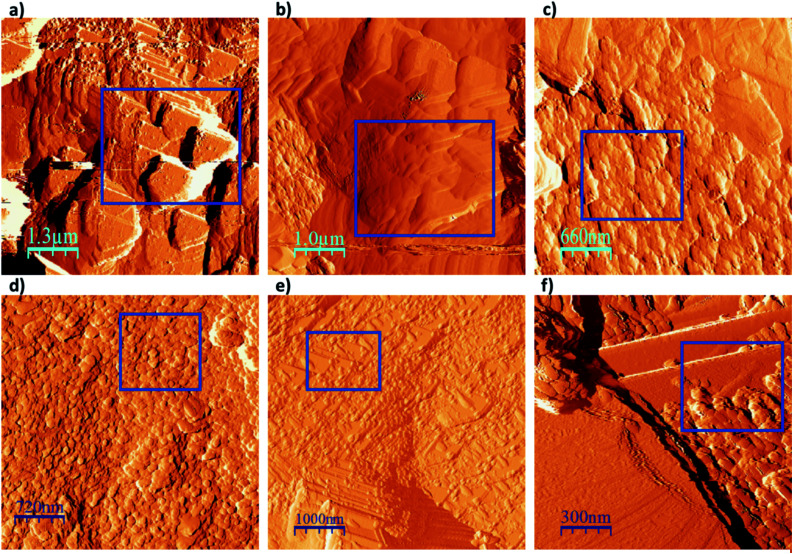
TM-AFM images of the SPCE when (a) unmodified, (b) following ANSA deposition, (c) following DNA attachment, (d) after hybridisation with miRNA, (e) after submersion in untreated urine and (f) in urine treated with proteinase K and spin filtration. Key observation areas are shown by blue boxes.

TM-AFM was then used to visualise the SPCE surface. As shown in Fig. 5, the following key steps in biosensor preparation were visualised: (a) the initial untreated SPCE surface, (b) the SPCE surface following ANSA deposition and (c) the SPCE sensor surface following DNA oligonucleotide attachment. Biosensors were then imaged after DNA/RNA hybridisation using (d) buffered synthetic miRNA, (e) untreated urine and (f) urine following proteinase K treatment and then filtration.

In Fig. 5a, clear triangular layered steps and terraces are observed in the topography of the carbon ink at the unmodified SPCE surface. The surface steps are sharp, with a typical length of 1.75 μm. Following electrodeposition of ANSA onto the SPCE surface these triangular layers can still be seen, but appear smoother and more rounded (Fig. 5b). Following DNA attachment to the SPCE surface *via* sulfonamide linkage (Fig. 5c), completing the biosensor structure, the image shows surface coverage by numerous small round artefacts approximately 40 nm in diameter, conceivably single-stranded DNA oligonucleotide clusters (Fig. 5c). A similar image is obtained following hybridisation with the target miRNA (Fig. 5d), with prevalent surface coverage by slightly larger round bodies of approximately 90 nm diameter.

The final two images relate to the biosensor surface following immersion in urine, one sample of which was unprocessed (Fig. 5e) and one sample which had been processed with proteinase K and membrane filtration (Fig. 5f). In Fig. 5e the round bodies described above that covered the biosensor surface were generally localised to the corners and edges of the triangular layers, and were larger than bodies observed in Fig. 5d, measuring approximately 120 nm in diameter. By contrast, Fig. 5f shows rounded bodies of approximately 80 nm located closer to the centre of the triangular terraces, closely resembling those seen in Fig. 5d.

These observations are consistent with our previous work, where unprocessed urine interfered with the electrochemical response of our glassy carbon biosensor to miRNA hybridisation.^24^ These findings provide strong evidence that the use of proteinase K and spin filtration removed contaminating lipids and macromolecules such as proteins and thereby prevented fouling of the biosensor surface. This ensured unimpeded biosensor miRNA hybridisation and accurate electrochemical output. Selected wider field images captured throughout the fabrication process are provided in the ESI (Fig. S14†).

## 4. Conclusions

In summary, we have demonstrated the utility of disposable SPCE-based biosensors for robust and highly sensitive miR-21 quantification in buffered solution. These biosensors were also able to replicate RT-qPCR data that discriminated between urine samples from DKD patients and unaffected control individuals on the basis of miR-192 expression relative to miR-191. We have therefore demonstrated that our disposable SPCE-based biosensors have potential for use in rapid urinary miRNA biomarker quantification. We have identified miRNA expression profiles associated with several renal and renal-related pathologies.^7–12^ The low cost and commercial availability of all materials used in our disposable sensor, along with its rapid production and short analysis time, make it highly advantageous to a financially pressed health service. With this in mind, we will now look to adapt our technologies for use in clinical testing. Future work will also investigate the simultaneous detection of multiple miRNAs on one sensor, as well as the triplicate reading usage presented here. Further enhancements of the sensor are being developed while investigating shelf life, storage conditions and use in different biological fluids including peritoneal dialysis effluent.

## Conflicts of interest

There are no conflicts to declare.

## Supplementary Material

RA-011-D0RA09874D-s001
