# Supplementary material for: Detection of urinary microRNA biomarkers using diazo sulfonamide-modified screen printed carbon electrodes
Source: RSC Adv. 2021 May 25;11(31):18832–9. doi: 10.1039/d0ra09874d (PMC8144888; doi:10.1039/d0ra09874d)
Supplement: RA-011-D0RA09874D-s001 [file RA-011-D0RA09874D-s001.pdf]

Electronic Supplementary Material (ESI) for RSC Advances.
This journal is © The Royal Society of Chemistry 2021

Detection of urinary microRNA biomarkers using diazo sulfonamide-modified screen printed carbon electrodes†

Electronic supplementary information

Daniel A. Smith[a,b], Kate Simpson[a], Matteo Lo-Cicero[c], Lucy J. Newbury[a,b], Philip Nicholas[d], Donald J. Fraser[a,b], Nigel Caiger[d], James E. Redman[b,c,1], Timothy Bowen[a,b,*,1]

[a] *Wales Kidney Research Unit, Division of Infection & Immunity, School of Medicine, College of Biomedical and Life Sciences, Cardiff University, Heath Park, Cardiff CF14 4XN, UK. E-mail: bowent@cf.ac.uk*
[b] *Cardiff Institute of Tissue Engineering and Repair, Museum Place, Cardiff CF10 3BG, UK*
[c] *School of Chemistry, College of Physical Sciences and Engineering, Cardiff University, Cardiff CF10 3AT, UK*
[d] *Sun Chemical Ltd, Midsomer Norton, Radstock, Bath BA3 4RT, UK*
[1] *These authors contributed equally to this work.*
**Corresponding author*

Table of Contents

Figure S1: Oligonucleotides used in the study. 2

Figure S2: CV changes over fabrication steps. 2

Figure S3: Reductive coulometry changes over fabrication steps. 3

Figure S4: Oxidative coulometry changes over fabrication steps. 3

Figure S5: CV deposition cycles. 4

Figure S6: Example oxidative coulometry response following hybridisation with decreasing concentrations of miR-21. 4

Figure S7: Oxidative coulometry calibration plot for miR-21. Data are expressed as mean +/- SEM. 5

Figure S8: Example differential pulse voltammetry (DPV) response following hybridisation with decreasing concentrations of miR-21. 5

Figure S9: Differential pulse voltammetry (DPV) calibration plot for miR-21. Data are expressed as mean +/- SEM. 6

Figure S10: Oxidative coulometry (Echem) and RT-qPCR (PCR) analysis of urinary miR-192 in DKD patients and control subjects. Data were normalised to miR-191 and are expressed as mean as +/- SEM (n = 6 each group). * = P < 0.05. 6

Figure S11: Reductive coulometry analysis of miR-21 in serial dilutions (1/10th, 1/50th, 1/100th, 1/500th, 1/1000th, 1/5000th) of control urine following proteinase K treatment. Data are expressed as mean +/- SEM (n = 3). 7

Figure S12: Reductive coulometry measurements investigating uric acid interference, uric acid solutions without microRNA in blue followed by addition of 10^{-11} M miR-21. Data are expressed as means +/- SEM (n = 3). 7

Figure S13: Oxidative coulometry measurements investigating uric acid interference, uric acid solutions without microRNA in blue followed by addition of 10^{-11} M miR-21. Data are expressed as means +/- SEM (n = 3). 8

Figure S14: Additional AFM images captured during SPCE modification. 9

Figure S1: Oligonucleotides used in the study.

**Oligonucleotide Name**	**Oligonucleotide Sequence**
comp-miR-21 (DNA)	5'NH_2-C_6-TCA ACA TCA GTC TGA TAA GCT A
miR-21 (RNA)	5'-UAG CUU AUC AGA CUG AUG UUG A
comp-miR-192 (DNA)	5'NH_2-C_6-GGC TGT CAA TTG ATA GGT CAG
miR-192 (RNA)	5'-CUG ACC UAU GAA UUG ACA GCC
comp-miR-191 (DNA)	5'NH_2-C_6-CAG CTG CTT TTG GGA TTC CGT TG
miR-191 (RNA)	5'-CAA CGG AAU CCC AAA AGC AGC UG
comp-miR-223 (DNA)	5'NH_2-C_6-TGG GGT ATT TGA CAA ACT GAC A
miR-16 (RNA)	5'-UAG CAG CAC GUA AAU AUU GGC G
3'-bio-comp-miR-21 (DNA)	5'NH_2-C_6-TCA ACA TCA GTC TGA TAA GCT A-Biotin
5'-bio-miR-21 (RNA)	5'Biotin-UAG CUU AUC AGA CUG AUG UUG A

*Comp = complementary, bio = biotin label.

Figure S2: CV changes over fabrication steps.

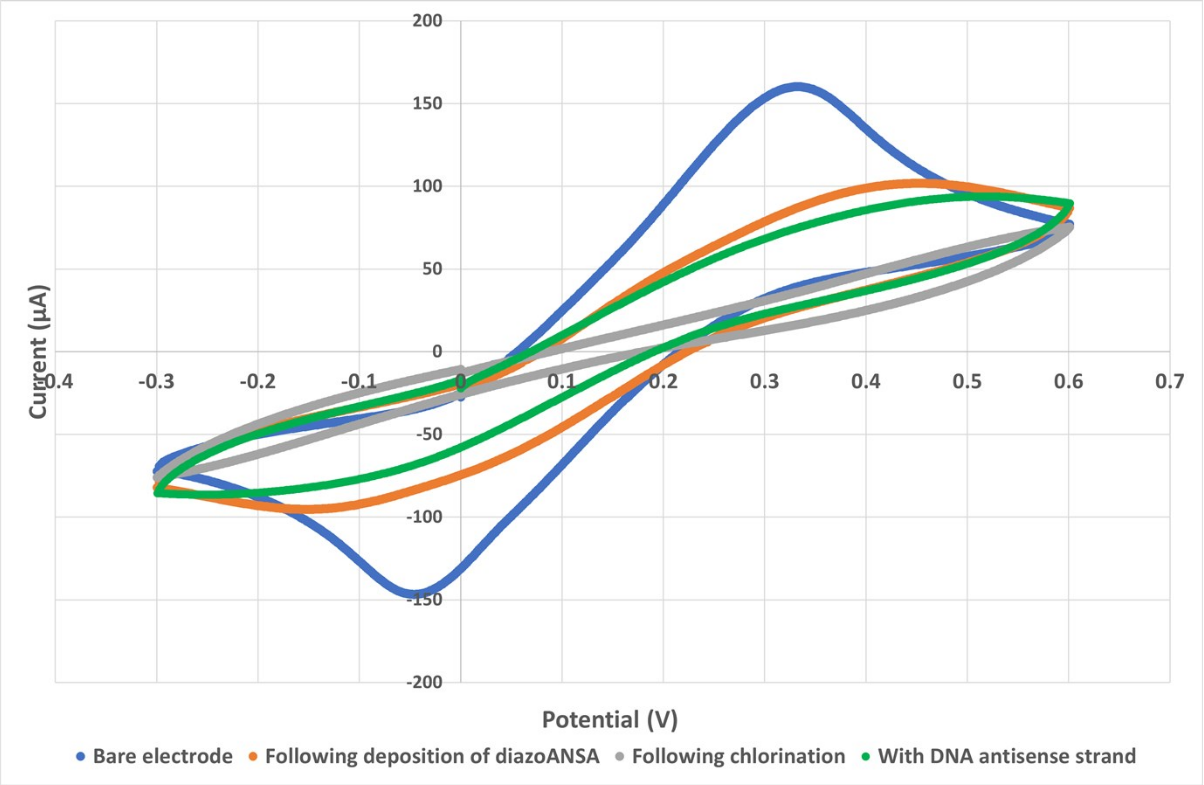

Figure S3: Reductive coulometry changes over fabrication steps.

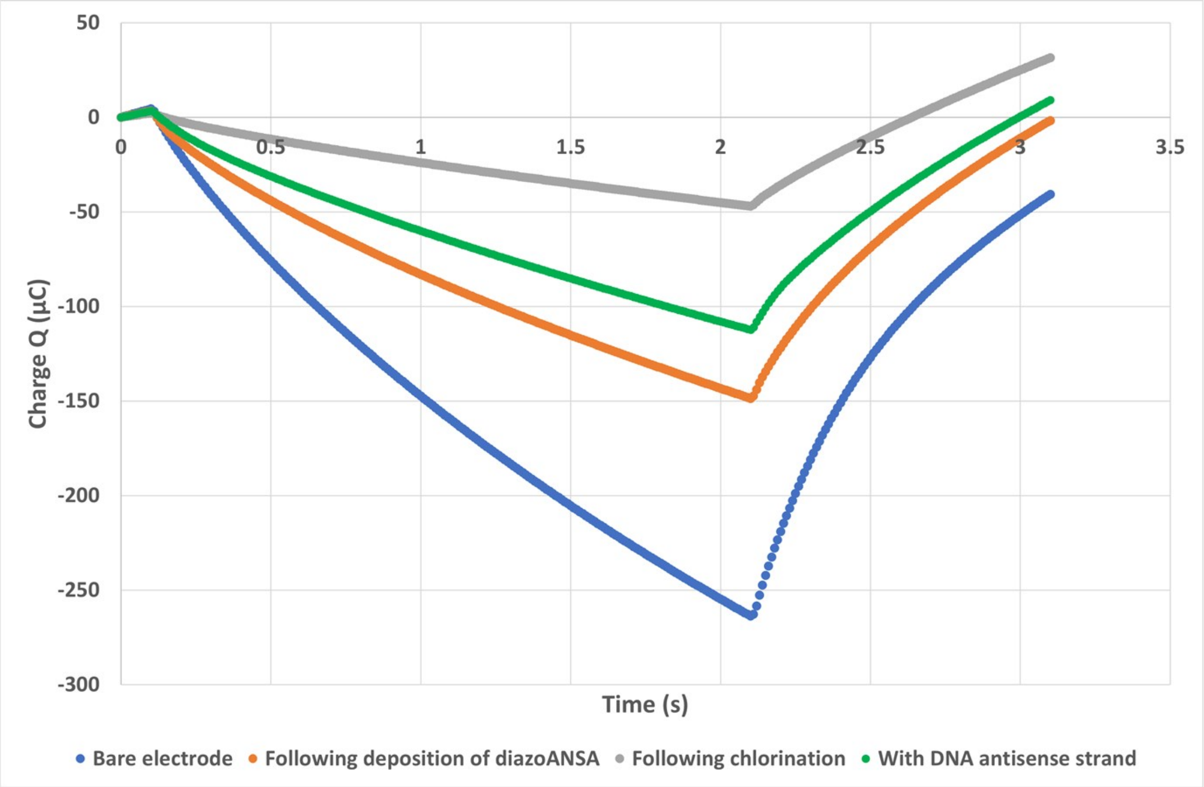

Figure S4: Oxidative coulometry changes over fabrication steps.

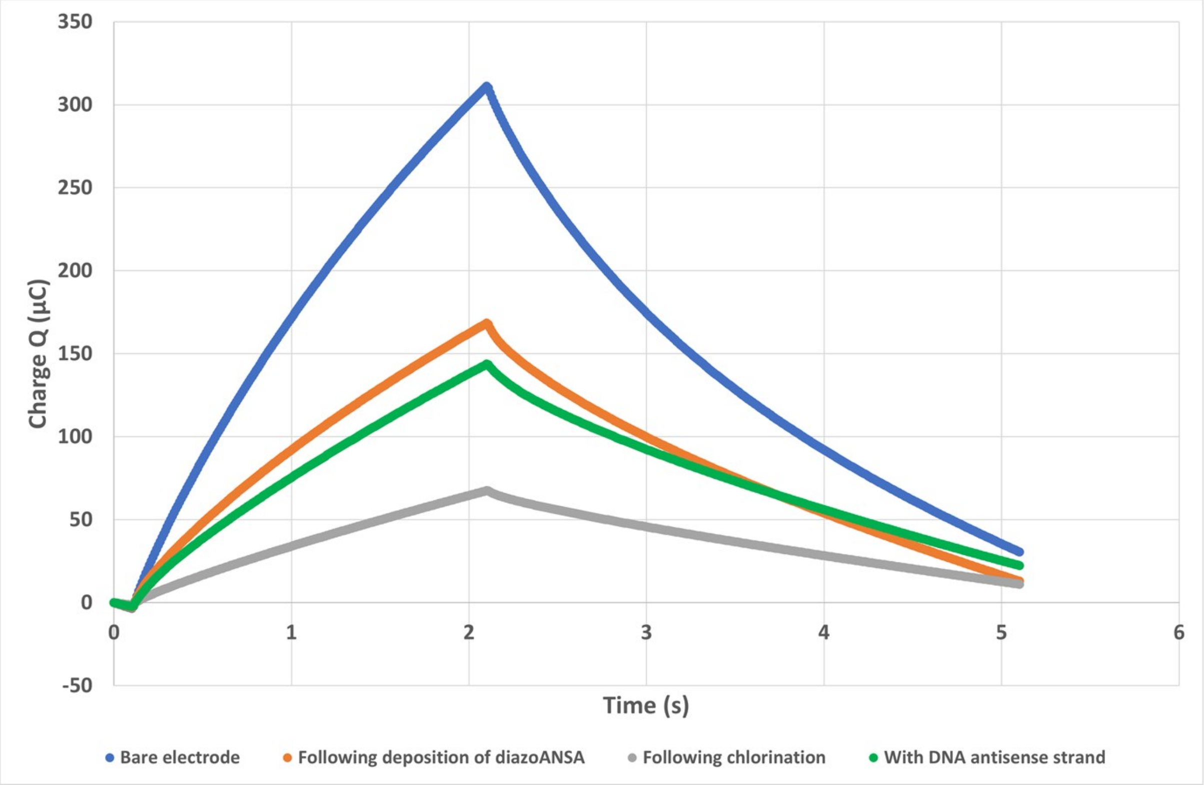

Figure S5: CV deposition cycles.

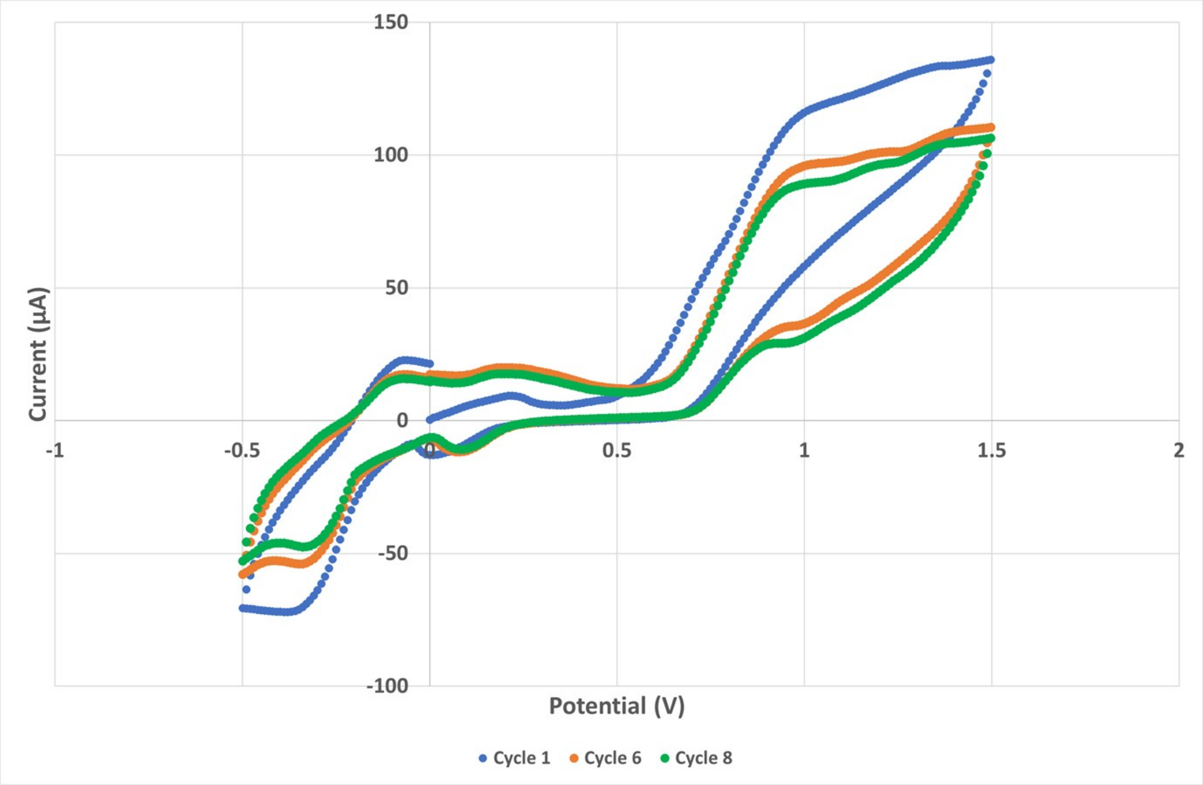

Figure S6: Example oxidative coulometry response following hybridisation with decreasing concentrations of miR-21.

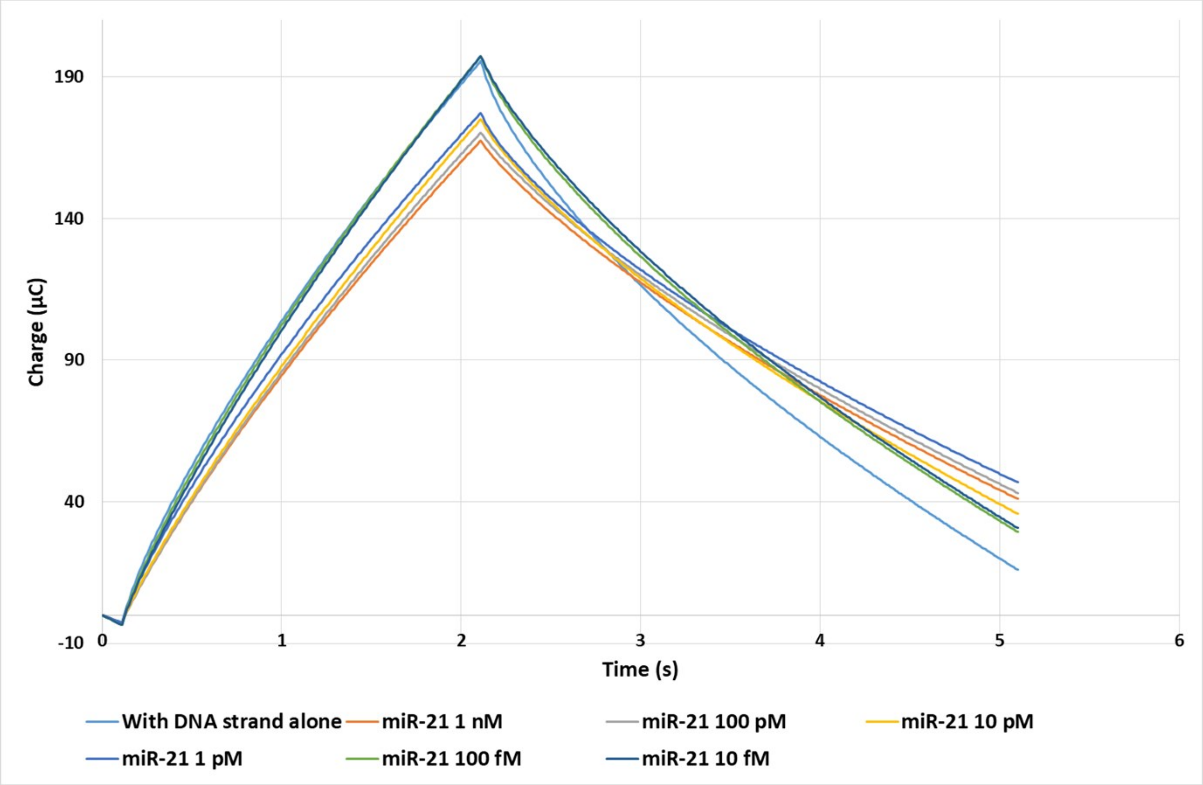

Figure S7: Oxidative coulometry calibration plot for miR-21. Data are expressed as mean +/- SEM (n = 11).

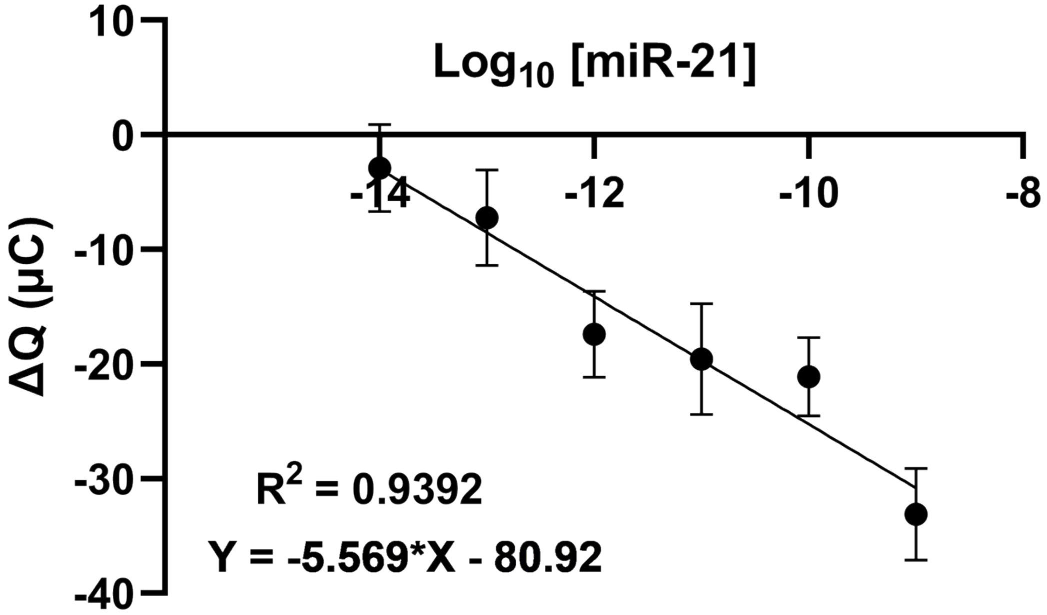

Figure S8: Example differential pulse voltammetry (DPV) response following hybridisation with decreasing concentrations of miR-21.

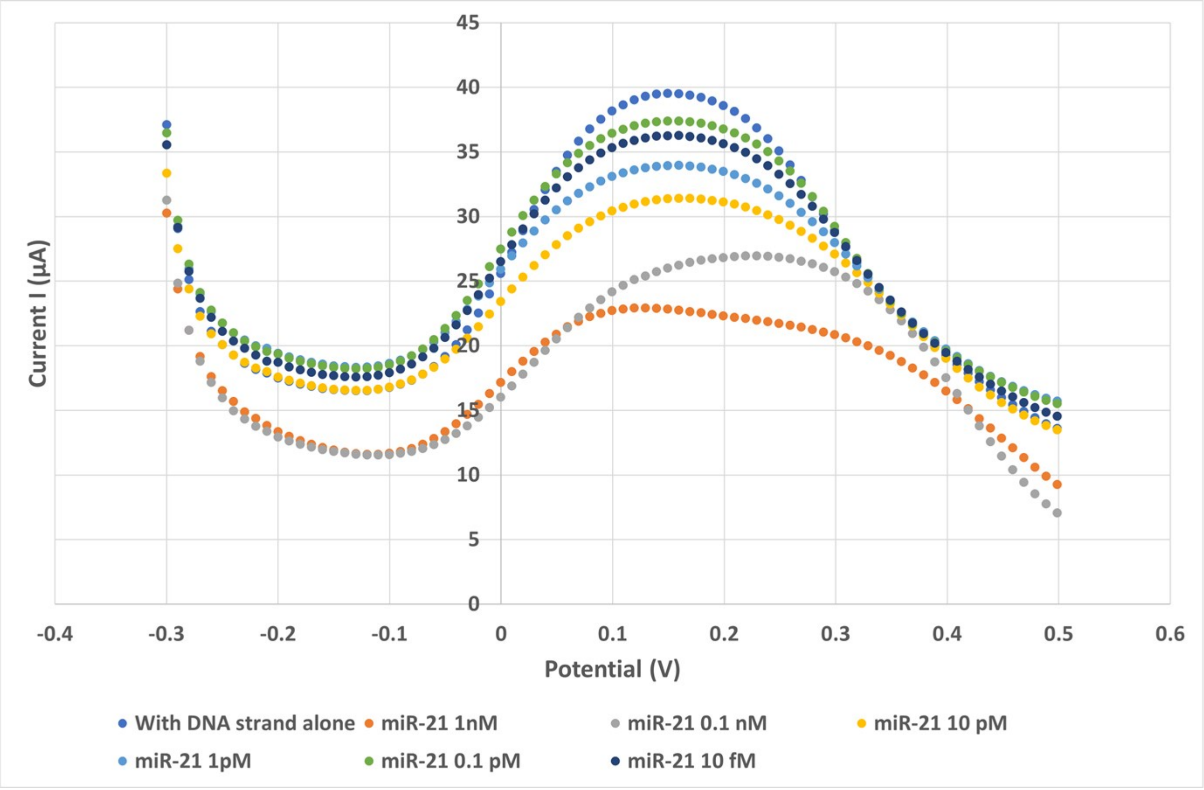

Figure S9: Differential pulse voltammetry (DPV) calibration plot for miR-21. Data are expressed as mean +/- SEM (n = 11).

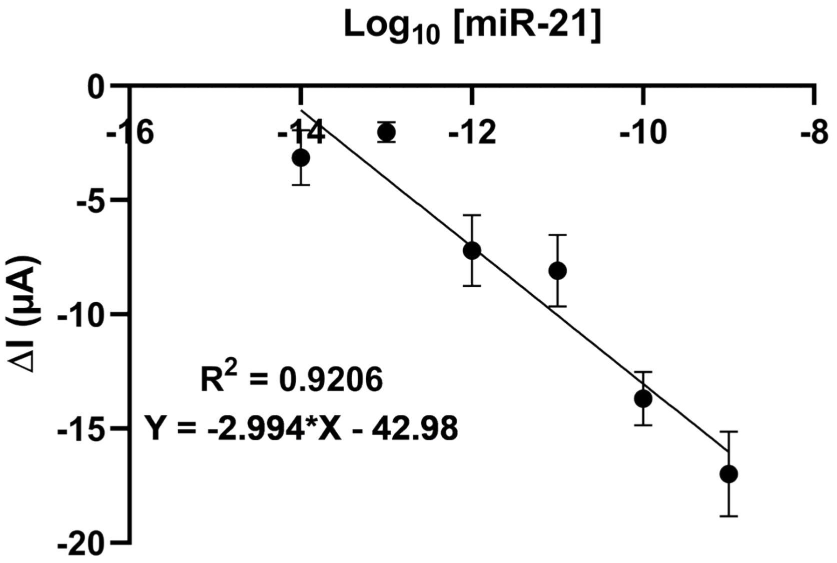

Figure S10: Oxidative coulometry (Echem) and RT-qPCR (PCR) analysis of urinary miR-192 in DKD patients and control subjects. Data were normalised to miR-191 and are expressed as mean as +/- SEM (n = 6 each group). * = P < 0.05.

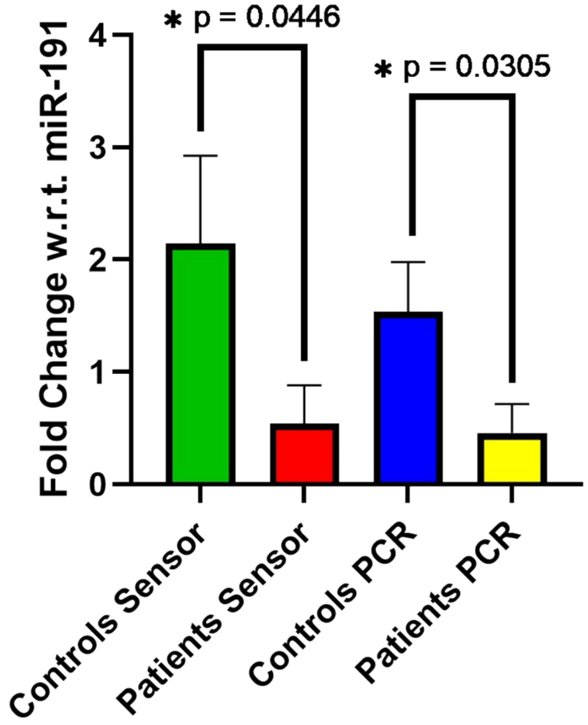

Figure S11: Reductive coulometry analysis of miR-21 in serial dilutions (1/10th, 1/50th, 1/100th, 1/500th, 1/1000th, 1/5000th) of control urine following proteinase K treatment. Data are expressed as mean +/- SEM (n = 3).

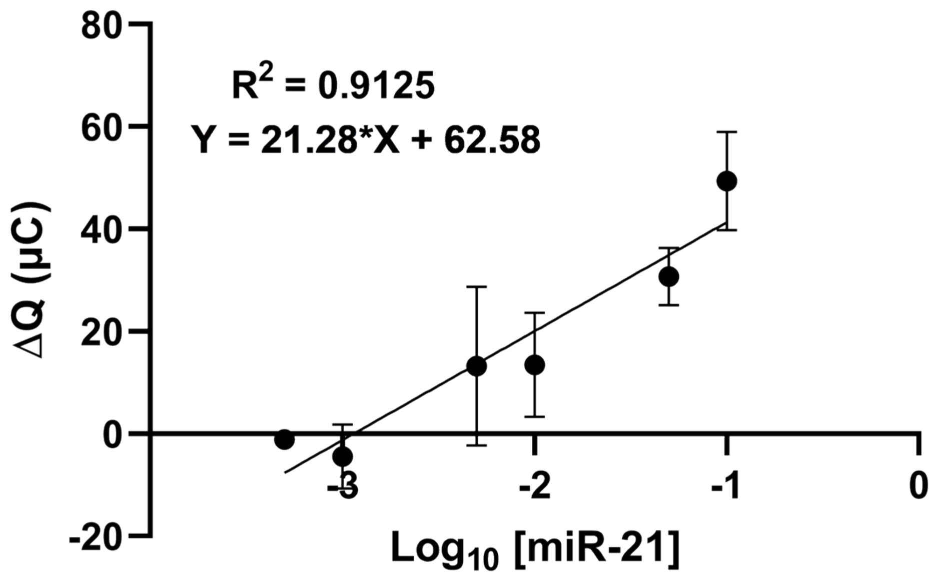

Figure S12: Reductive coulometry measurements investigating uric acid interference, uric acid solutions without miR-21 in blue followed by addition of 10^{-11} M miR-21. Data are expressed as mean +/- SEM (n = 3).

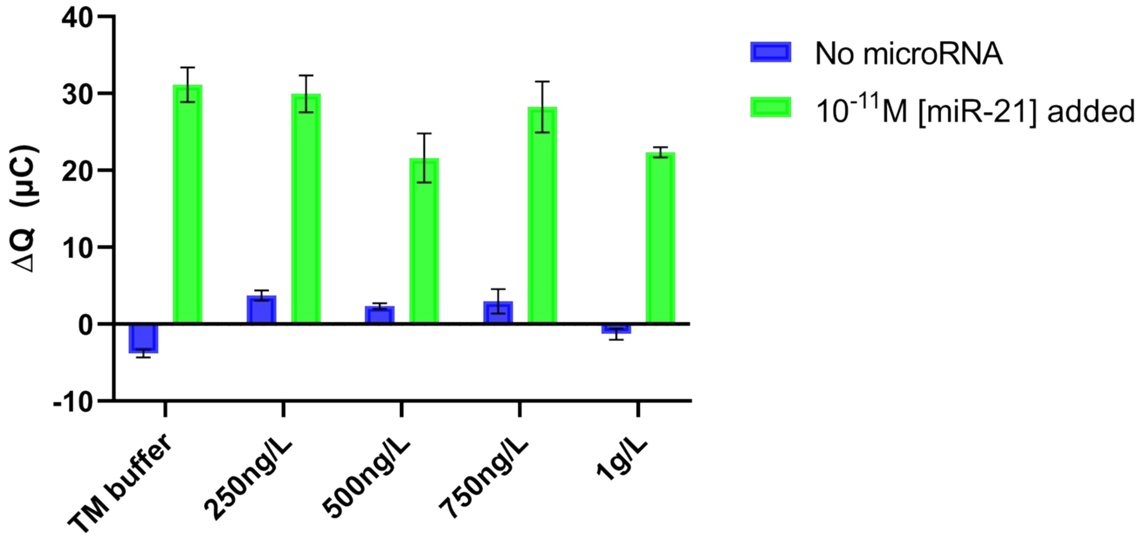

Figure S13: Oxidative coulometry measurements investigating uric acid interference, uric acid solutions without miR-21 in blue followed by addition of 10^{-11} M miR-21. Data are expressed as mean +/- SEM (n = 3).

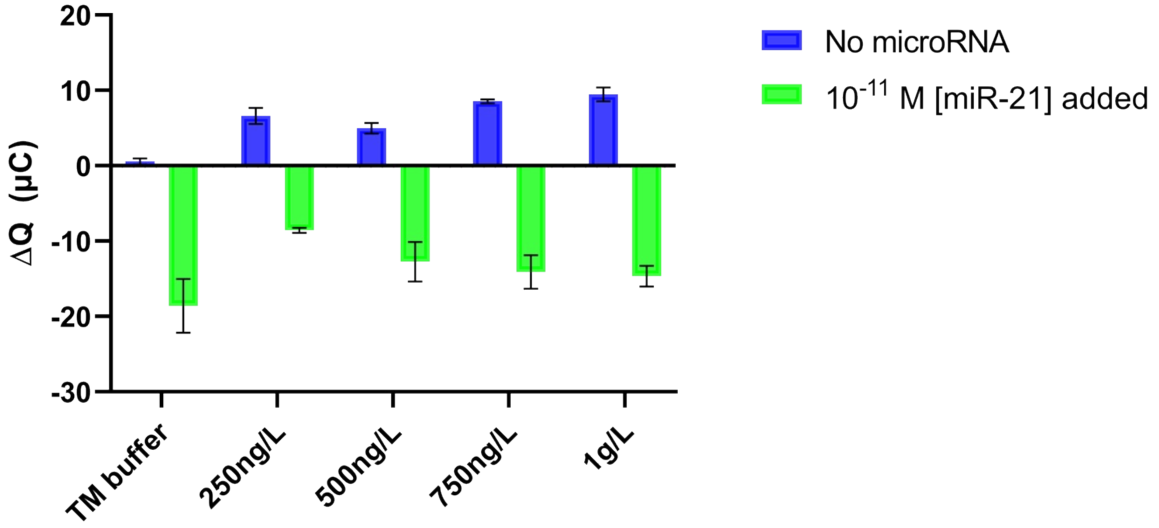

Figure S14: Additional TM-AFM images captured during SPCE modification.

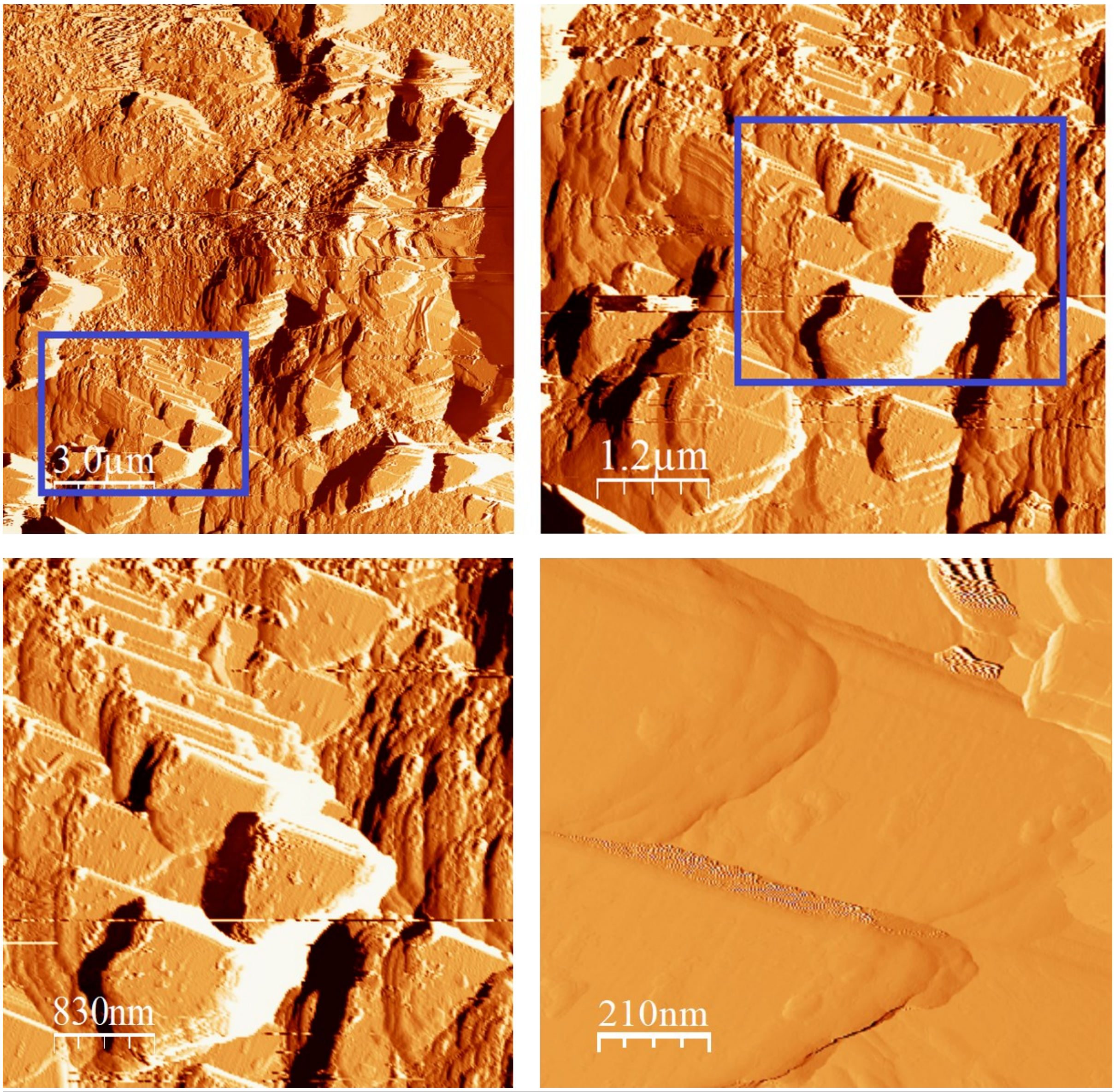

Fig. S14-1 TM-AFM images of untreated SPCE surface, blue boxes highlight areas of increased magnification.

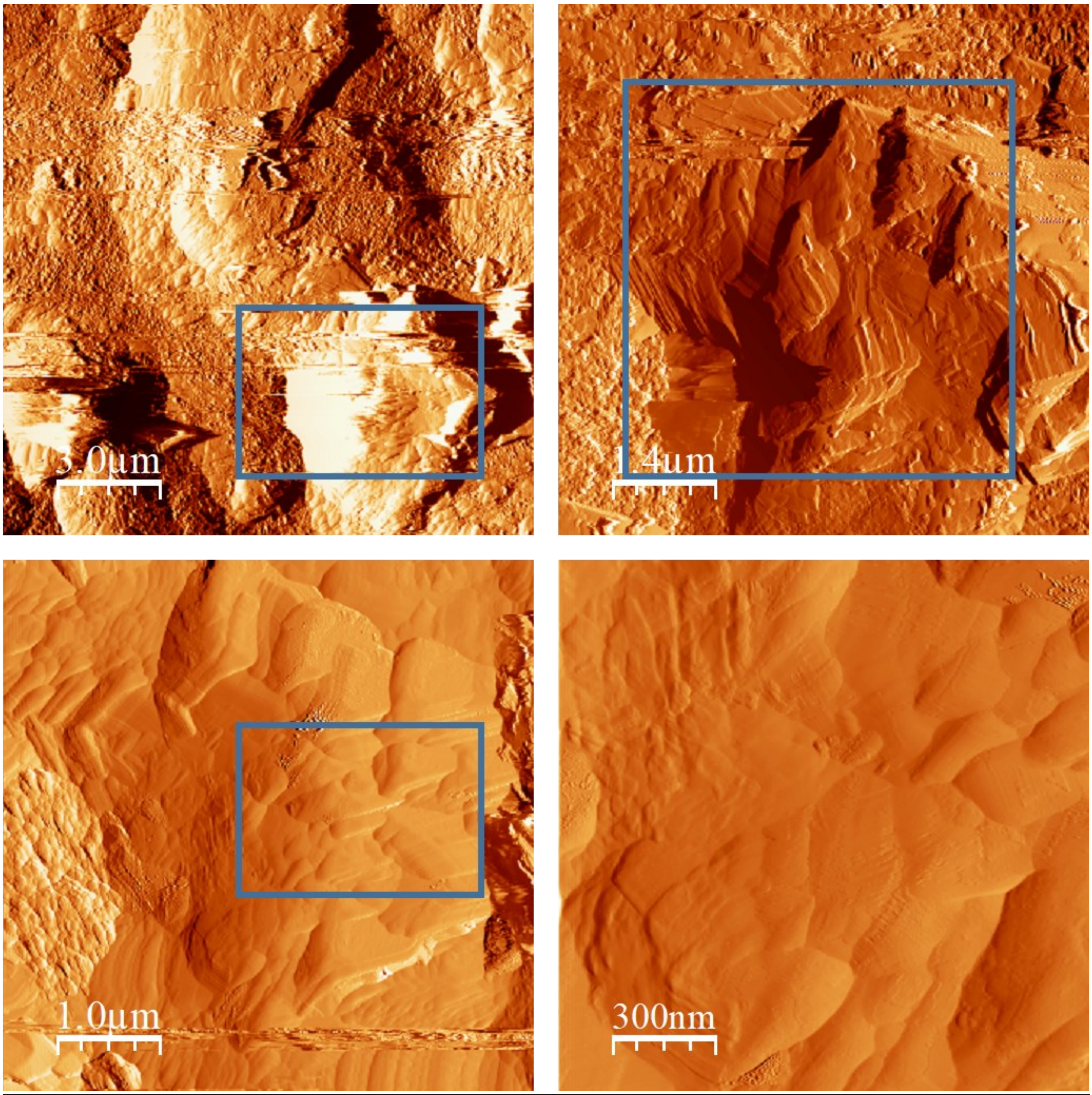

Fig. S14-2 TM-AFM images of the SPCE surface following ANSA deposition, blue boxes highlight areas of increased magnification.

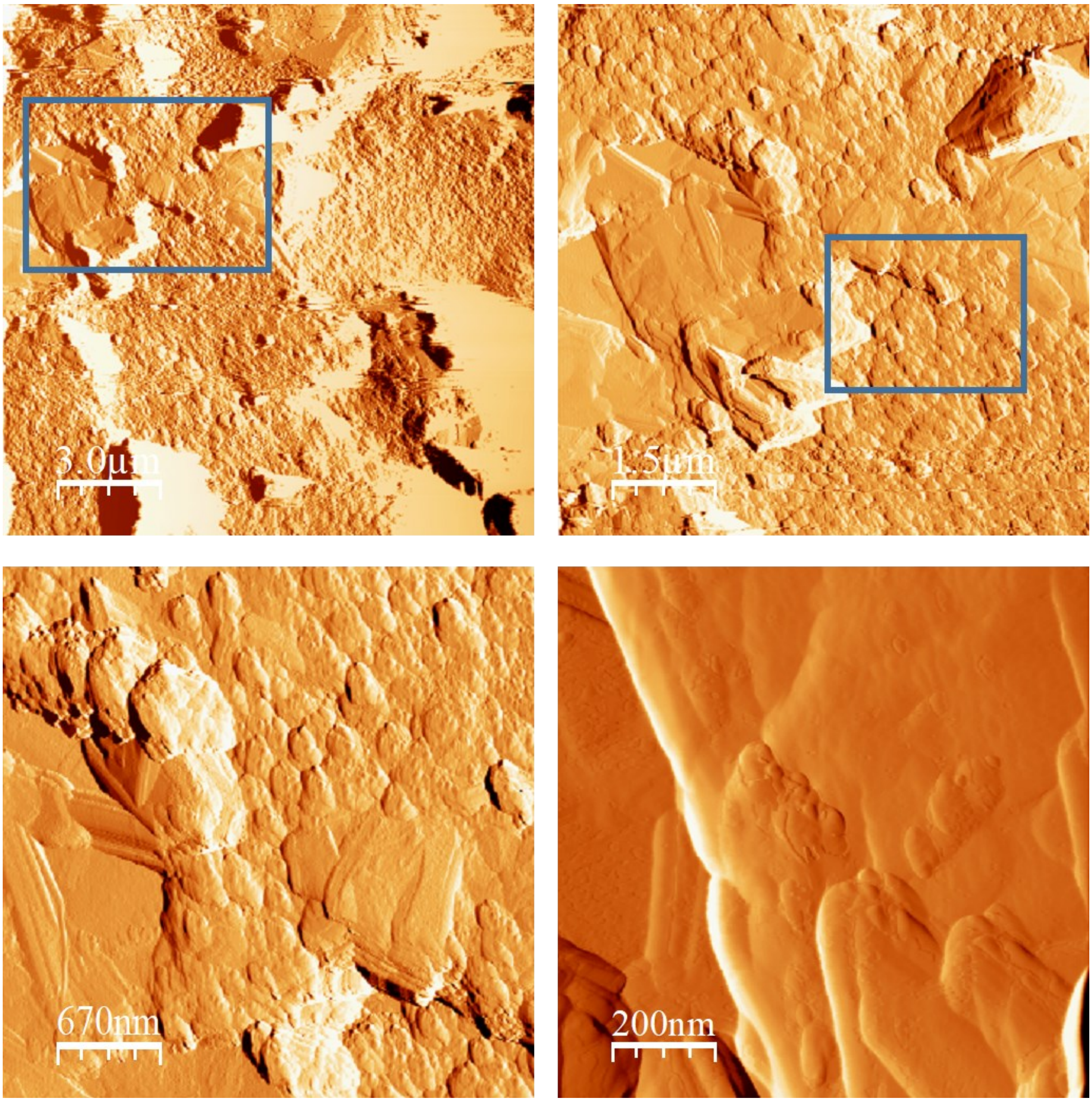

Fig. S14-3 TM-AFM images of the SPCE surface following DNA oligonucleotide attachment, blue boxes highlight areas of increased magnification.

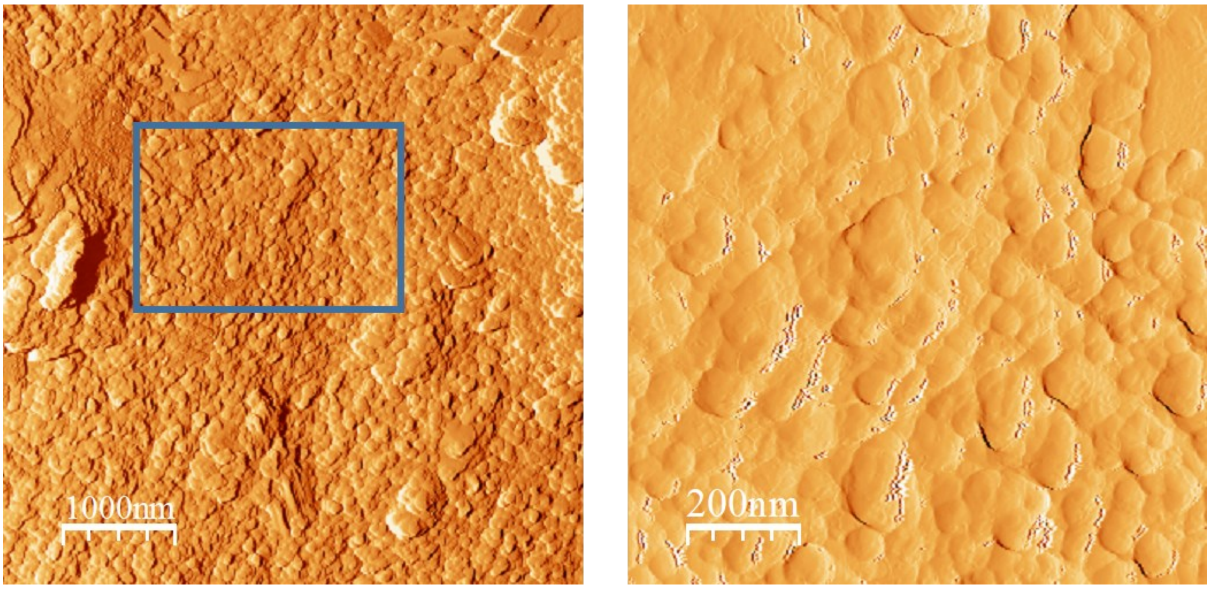

Fig. S14-4 TM-AFM images of the SPCE biosensor surface following miRNA hybridisation, blue box highlights area of increased magnification.

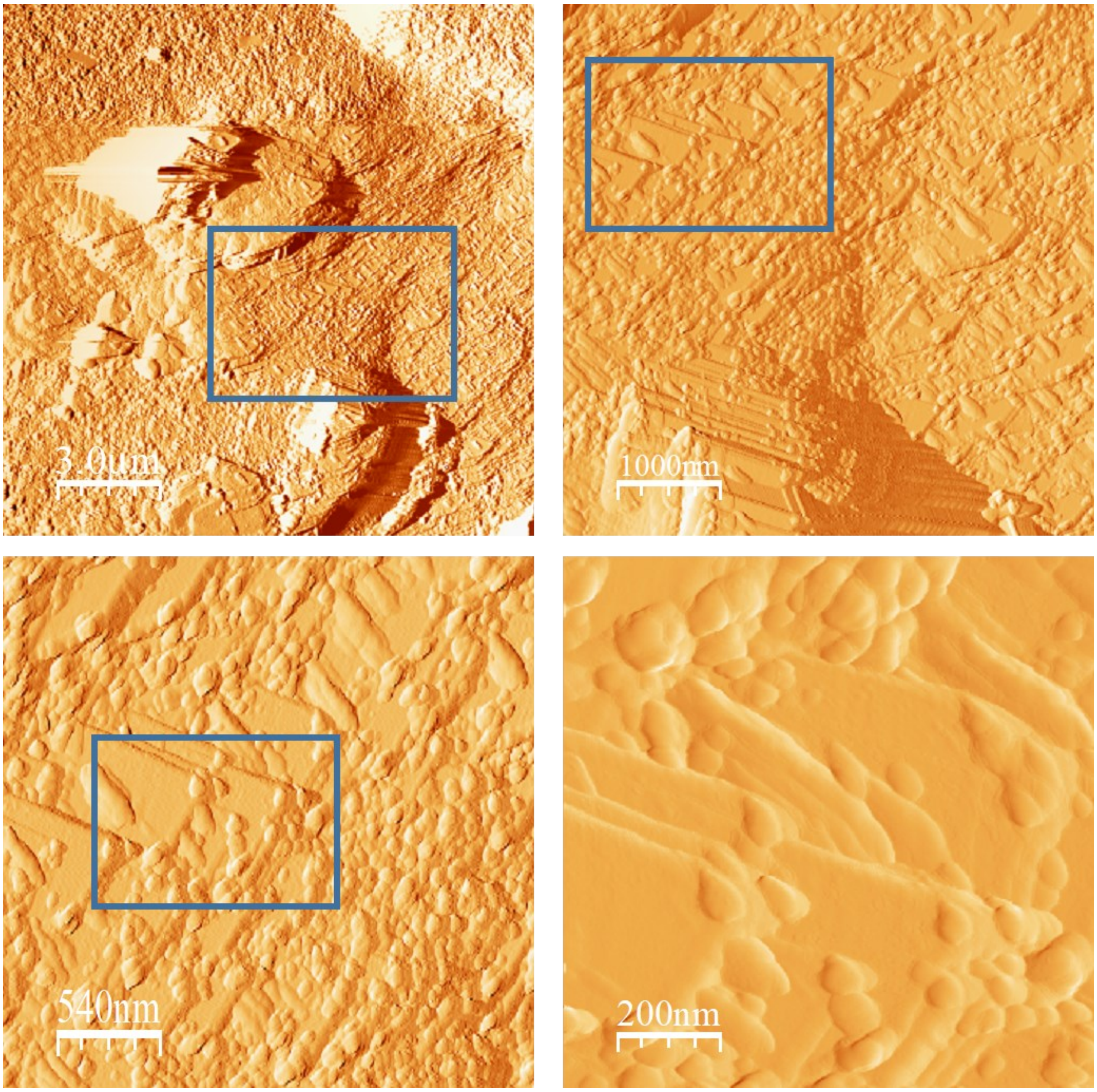

Fig. S14-5 TM-AFM images of the SPCE biosensor surface following exposure to unprocessed urine, blue boxes highlight areas of increased magnification.

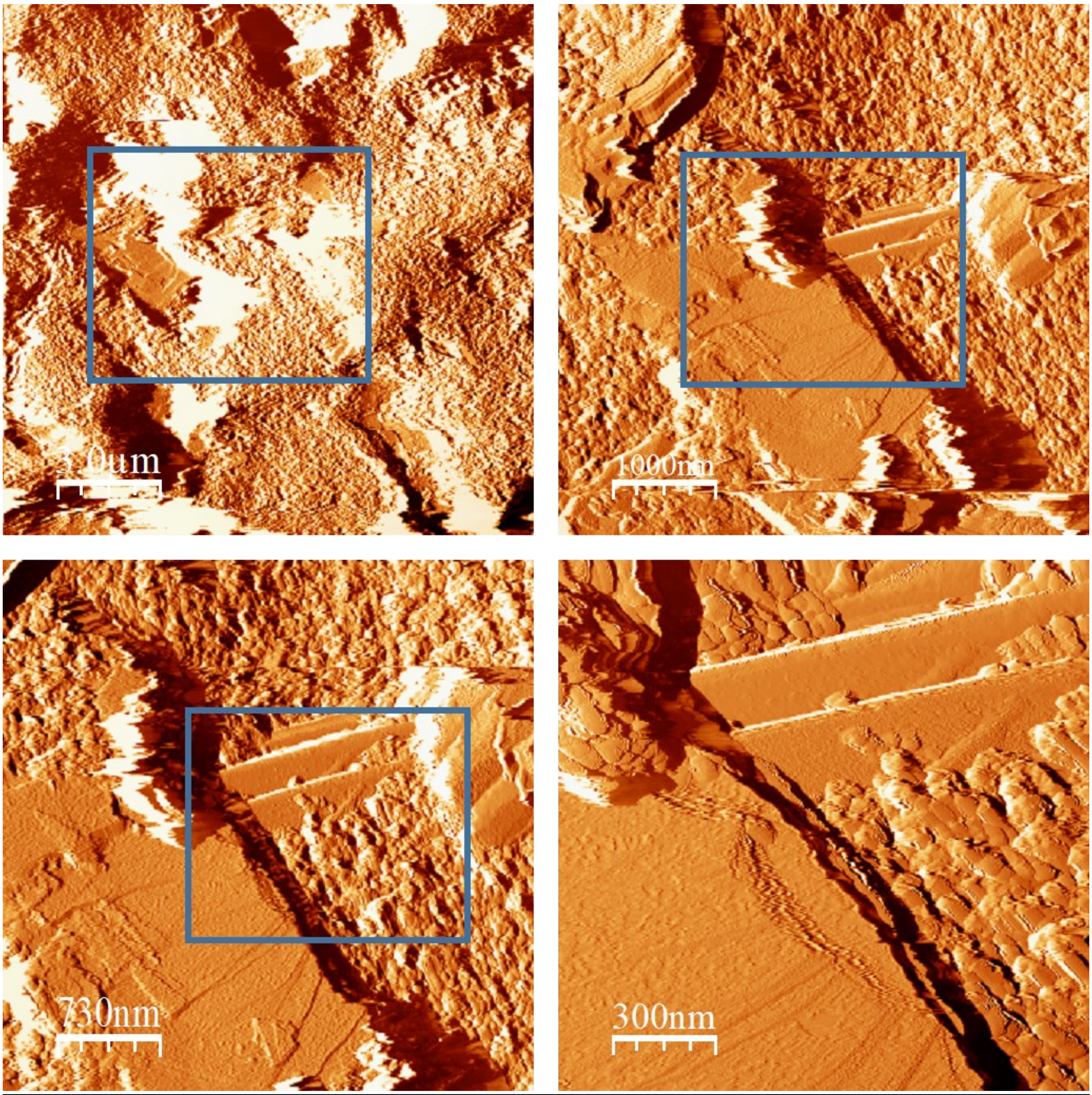

Figure S14-6 TM-AFM images of the SPCE biosensor surface following exposure to proteinase K treated urine, blue boxes highlight areas of increased magnification.
